# Assessment of Myocardial Performance Index and Aortic Elasticity in Patients With Beta-Thalassemia Major

**DOI:** 10.14740/jocmr2293w

**Published:** 2015-08-23

**Authors:** Cem Sahin, Ozcan Basaran, Ibrahim Altun, Fatih Akin, Yasar Topal, Hatice Topal, Murat Biteker, Mehmet Fatih Azik

**Affiliations:** a Department of Internal Medicine, Faculty of Medicine, Mugla Sitki Kocman University, Mugla, Turkey; b Department of Cardiology, Faculty of Medicine, Mugla Sitki Kocman University, Mugla, Turkey; c Department of Pediatrics, Faculty of Medicine, Mugla Sitki Kocman University, Mugla, Turkey

**Keywords:** Beta-thalassemia, Myocardial performance index, Aortic elasticity

## Abstract

**Background:**

This study aimed to assess myocardial performance index (MPI) and arterial elasticity indices in asymptomatic patients with beta-thalassemia major without known heart disease and to determine relationship between these indices and parameters indicating iron load of body.

**Methods:**

The study included 55 asymptomatic beta-thalassemia patients (median age: 20 years (10 - 48 years)) without known history of heart disease and 40 age- and sex-matched healthy controls. MPI and arterial elasticity indices were determined by using standard two-dimensional and Doppler echocardiography. Data were analyzed by SPSS for Windows version 20.0 (SPSS Inc., Chicago, IL, USA).

**Results:**

Left ventricular mass index (83.917 (50.62 - 144) and 68.37 (41.9 - 113.3)) and MPI (0.464 (0.33 - 0.68) and 0.431 (0.31 - 0.51)) were significantly higher in patients with beta-thalassemia when compared to control group (P < 0.001 and P = 0.006). Aortic elasticity indices were significantly higher while aortic strain and distensibility values were significantly lower in patients with beta-thalassemia compared to controls (all P values < 0.001). Positive correlations were detected between aortic stiffness index and platelet (r = 0.235; P = 0.019) and ferritin values (r = 0.328; P = 0.008). Presence of thalassemia (β = -0.729; P = 0.041) and higher platelet value (β = 0.235; P = 0.019) were significant determinants for increased aortic stiffness in linear regression analysis.

**Conclusion:**

Arterial elasticity indices and MPI are impaired in patients with beta-thalassemia major and these parameters may be used to predict cardiovascular complications in asymptomatic patients with beta-thalassemia major.

## Introduction

Thalassemia is a group of heterogeneous diseases with autosomal recessive inheritance, which is characterized by hypochromic, microcytic anemia resulting from disrupted synthesis of hemoglobin chains. Degree of cardiovascular involvement is one of the major factors determining prognosis in beta-thalassemia syndromes. Parenchymal injury secondary to myocardial iron deposition and immune-inflammatory processes are known to be primary reasons for cardiovascular disorders in patients with thalassemia [1]. It is known that early abnormalities in ventricular myocardium can occur in patients with beta-thalassemia, even in those receiving effective chelation therapies. In addition to parenchymal injury, vascular pathologies also facilitate cardiovascular complications in patients with thalassemia [2].

Echocardiography is a reliable, feasible and non-invasive modality for early detection and serial assessment of alterations in cardiac morphology and functions in patients at risk for myocardial iron deposition. Myocardial performance index (MPI) and arterial elasticity indices are helpful for early diagnosis of cardiovascular complications [3]. In previous studies, it was shown that these indices can be used to detect cardiovascular pathologies in patients with thalassemia even if classical echocardiography parameters such as ejection fraction are normal [4]. There are limited number of studies investigating the role of both MPI and arterial stiffness indices in patients with beta-thalassemia major. In this study, we aimed to assess MPI and arterial elasticity indices in asymptomatic beta-thalassemia patients without known heart disease and to determine relationship between these indices and parameters indicating iron load of body.

## Material and Method

In this case-control study, 55 patients (median age: 20 years (10 - 48 years)) suffering from B-thalassemia major were recruited from the Thalassemia Clinic of Mugla Sitki Kocman University Education and Research Hospital. Patients with heart failure, systemic hypertension, diabetes mellitus, thyroid dysfunction, or parathyroid dysfunction were excluded. They had normal echocardiography and were on sinus rhythm. Forty age- and sex-matched healthy individuals, with no clinical, electrocardiographic, or echocardiographic evidence of cardiovascular disease were enrolled as controls. The Local Ethics Committee approved the study, and all subjects gave informed consent.

### Study design

All patients underwent a thorough clinical, electrocardiographic, and echocardiographic examination 48 h prior to blood transfusion, and blood samples were collected for hematologic measurements on the same day. The control group also underwent a full echocardiographic study and blood measurements. Body weight and height were measured, and body surface area was calculated accordingly.

### Blood pressure (BP) measurement

BP levels were measured from the brachial artery at the level of the heart with a sphygmomanometer after resting for at least 5 min in the supine position. Three measurements, at least 2 min apart, were carried out, and the average of the closest two readings was recorded. A pressure drop rate of approximately 2 mm Hg/s was applied, and Korotkoff phases I and V were used for systolic and diastolic BP levels, respectively. All BP measurements were made by a cardiologist blinded to the study protocol. Pulse pressure (PP) was calculated as systolic minus diastolic BP.

### Echocardiography

Standard two-dimensional and Doppler echocardiography was performed using a commercially available echocardiographic machine (Vivid S6 GE Medical System, Horten, Norway) with a 2.0 - 3.5 MHz transducer. Measurements were made during normal breathing at end expiration. Left ventricular (LV) end-systolic (LVSd) and end-diastolic diameters (LVDd), end-diastolic interventricular septal thickness (IVSd) and end-diastolic LV posterior wall thickness (PWd) were measured at end-diastole according to established standards of the American Society of Echocardiography [5]. LV mass (LVM) was calculated using the Devereux formula: 0.8(1.04(((LVDd + IVSd + PWd)3 - LVDd3))) + 0.62. Thereafter, LV mass index (LVMi) was obtained by the following formula: LVM/body surface area [6].

### Standard and tissue Doppler echocardiography

Pulsed Doppler ultrasound scanning recordings of the mitral inflow velocities were obtained from the apical four-chamber view by placing the sample volume between the tips of the mitral leaflets. Peak early (E) and late (A) diastolic velocities, deceleration time, and the ratio of early to late peak velocities (E/A), were analyzed. Pulse wave tissue Doppler was performed with a 2-mm sample volume placed at the lateral corner of the mitral annulus from the apical four-chamber view. Filters were set to exclude high-frequency signals and gain minimized. Doppler ultrasound scanning intervals were measured from mitral annular velocity intervals. Left ventricular MPI was determined by using this equation: MPI = (IRT + ICT)/ET, where IRT is isovolumic relaxation time, ICT is isovolumic contraction time, and ET is ejection time [7].

### Echocardiography of the aorta

**Figure 1:**
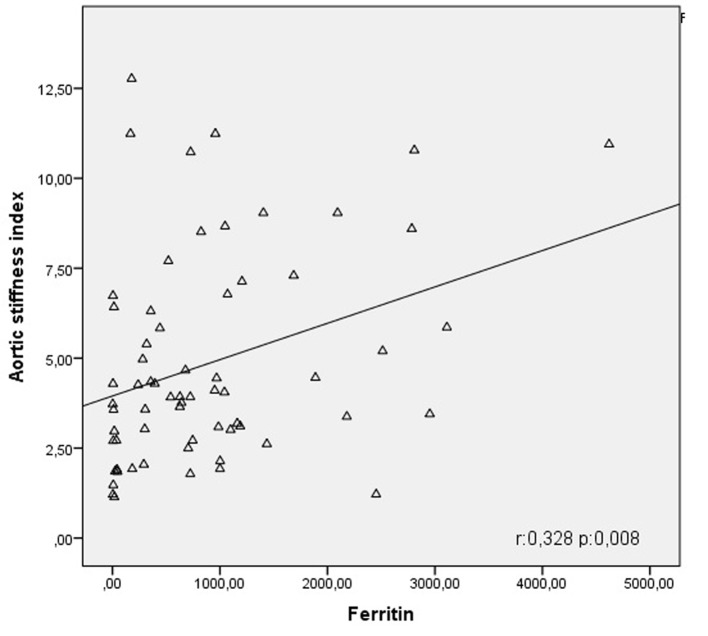
Correlation curve between aortic stiffness index and ferritin.

Thoracic aortic diameters (cm) were measured 3 cm above the aortic valve by two-dimensional guided M-mode transthoracic echocardiography of the aortic root at left parasternal long-axis view (Fig. 1). Aortic systolic diameter (AoS) was measured at the time of full opening of the aortic valve, and diastolic diameter (AoD) at the peak of the QRS complex at the simultaneous electrocardiogram recording. Inner aortic diameters were measured with a caliper in systole and diastole as the distance between the trailing edge of the anterior aortic wall and the leading edge of the posterior aortic wall. Measurements were repeated at three cardiac cycles and the averaged value was used for analysis. All echocardiographic measurements were made by a cardiologist blinded to the study protocol. The elasticity of the aorta was assessed by the following formulas: Aortic strain (%) = (AoS - AoD) × 100/AoD; aortic distensibility (cm^2^dyn-110-3) = (2 × aortic strain)/PP; aortic stiffness index = (ln (systolic pressure/diastolic pressure))/aortic strain; and elastic modulus E(p) = PP/strain [8]. Stiffness index is a marker for aortic stiffness, whilst strain, distensibility and elastic modulus are markers for aortic elasticity. These parameters give information on structure of aorta. Abnormality in these parameters means structural alteration of arterial wall. Elastic properties of aorta are useful not only in representing basic mechanical behavior of the arterial system but also in predicting outcome.

### Statistical analysis

Data were analyzed by using SPSS for Windows version 20.0 (SPSS Inc., Chicago, IL, USA). Kolmogorov-Smirnov test was used to assess distribution of continuous variables where homogeneity was tested. Numeric variables with normal distribution were expressed as mean ± standard deviation while those with skewed distribution were expressed as median (min-max). Log transformation was applied to numeric variables with skewed distribution. After log transformation, it was seen that parameters including age, E/A ratio, RDW, lymphocyte values, aortic strain, distensibility, stiffness and elastic modulus exhibited normal distribution pattern. Student’s *t*-test was used to compare mean values of quantitative variables with normal distribution while Mann-Whitney U test was used to compare quantitative variables with skewed distribution. In correlation analyses, Pearson test was used for parametric variables while Spearmen test was used for non-parametric variables. Linear regression analysis was performed to assess relationship between variables found to be correlated. Statistical significance was interpreted by using P value in data obtained. P value < 0.05 was considered as statistically significant.

## Results

Table 1 summarizes clinical, hematological and echocardiographic parameters in the thalassemia and control groups. It was found that there were significant differences in systolic and diastolic BPs, E/E’ ratio, left atrium diameter, DTE, hemoglobin, platelet counts and ferritin levels while no significant difference was detected in echocardiographic parameters including EF, E/A ratio, RWT and MAPSE values between the thalassemia and control groups. It was found that BP values and hemoglobin values were lower while ferritin and platelet values were higher in thalassemia group when compared to controls. E/E’ ratio, marker of diastolic function, and DTE values were found to be significantly higher in thalassemia group compared to controls.

**Table 1:** Clinical, Hematological and Echocardiographic Parameters of Cases

	Thalassemia n (range)	Control	P
Age	20 (10 - 48)	20 (10 - 42)	0.808
Gender			
Male, % (n)	58.7 (27)	41.3 (19)	0.558
Female, % (n)	52.8 (28)	47.2 (25)	
Systolic blood pressure	100 (90 - 130)	110 (120 - 140)	< 0.001
Diastolic blood pressure	60 (50 - 70)	70 (60 - 90)	< 0.001
Pulse pressure	30 (30 - 60)	40 (25 - 60)	0.187
EF (%)	69 (53 - 79)	68 (60 - 83)	0.434
E/A ratio	1.957 ± 0.719	1.842 ± 0.666	0.418
E/E’ ratio	7.058 ± 2.15	6.076 ± 1.824	0.018
RWT	0.391 ± 0.067	0.372 ± 0.061	0.161
LA diameter (cm)	3.129 ± 0.458	2.761 ± 0.521	< 0.001
DTE (msn)	184.2 ± 49.261	214.16 ± 49.61	0.004
MAPSE	1.6 (1.2 - 1.9)	1.5 (1.2 - 1.8)	0.157
Hemoglobin	8.4 (6.5 - 10)	13.2 (12 - 16.2)	< 0.001
Platelet	398 (115 - 976)	247 (132 - 440)	< 0.001
Ferritin	785 (13 - 4,020)	25 (8 - 145)	< 0.001

Table 2 summarizes myocardial and aortic elasticity indices in thalassemia and control groups. It was seen that left ventricular mass index was significantly higher in patients with thalassemia compared to controls (P < 0.001). Similarly, it was seen that MPI was higher in patients with thalassemia compared to controls (P = 0.006). When aortic elasticity parameters were considered, it was seen that aortic stiffness index and elastic modulus were significantly higher, while aortic strain and distensibility values were lower in thalassemia group compared to controls (P < 0.001).

**Table 2:** Results of Myocardial Performance Index and Aortic Elasticity Parameters in Thalassemia and Control Groups

	Thalassemia	Control	P
LV mass index	83.917 (50.62 - 144)	68.37 (41.9 - 113.3)	< 0.001
Myocardial performance index (g/m^2^)	0.464 (0.33 - 0.68)	0.431 (0.31 - 0.51)	0.006
Aortic strain	10.526 (3.7 - 33.3)	14.642 (7.69 - 35.29)	< 0.001
Aortic distensibility	5.797 (2 - 22.2)	8.333 (3.2 - 20.83)	< 0.001
Aortic stiffness index	4.257 (1.22 - 12.77)	2.775 (1.14 - 6.74)	< 0.001
Elastic modulus	3.45 (0.9 - 10)	2.4 (0.96 - 6.25)	< 0.001

Table 3 summarizes results of correlation and regression analysis between aortic stiffness index and clinical and hematological parameters. In our study, a negative correlation was detected between aortic stiffness index and thalassemia major (r = -0.443; P < 0.001) (Fig. 1). Similarly, it was seen that aortic stiffness index was negatively correlated with hemoglobin levels (r = -0.375; P < 0.001) while it was positively correlated with platelet (r = 0.235; P = 0.019) and ferritin values (r = 0.328, P = 0.008) (Fig. 1). When thalassemia, hemoglobin, platelet value and ferritin level found to be correlated to aortic stiffness index were subjected to linear regression analysis, it was shown that only thalassemia (β = -0.729; P = 0.041) and platelet value (β = 0.235; P = 0.019) were significant determinants for aortic stiffness index.

**Table 3:** Bivariate Correlation and Multiple Linear Regression Analysis Between Aortic Stiffness Index and Other Variables

Parameters	Bivariate analysis		Multiple linear regression analysis	
	r	P	Beta	P
Age	0.022	0.830		
BMI	-0.077	0.447		
Gender	-0.092	0.364		
Thalassemia	-0.443	< 0.001	-0.729	0.041
Systolic BP	-0.040	0.691		
Diastolic BP	0.205	0.042	0.164	0.154
Hemoglobin	-0.375	< 0.001	0.488	0.163
PLT	0.235	0.019	0.235	0.019
Ferritin	0.328	0.008	0.216	0.082

Table 4 summarizes results of correlation and regression analysis between aortic strain and clinical and hematological parameters. In our study, a positive correlation was detected between aortic stiffness index and thalassemia major (r = 0.387; P < 0.001). Similarly, it was seen that aortic strain was positively correlated with hemoglobin levels (r = 0.324; P = 0.048) while it was negatively correlated with platelet count (r = -0.119; P = 0.048) and ferritin values (r = -0.298, P = 0.016). When thalassemia, hemoglobin, platelet value and ferritin level found to be correlated to aortic strain were subjected to linear regression analysis, it was shown that only thalassemia (β = 0.783; P = 0.032) and platelet value (β = -0.199; P = 0.048) were significant determinants for aortic stiffness index.

**Table 4:** Bivariate Correlation and Multiple Linear Regression Analysis Between Aortic Strain and Other Variables

Parameters	Bivariate analysis		Multiple linear regression analysis	
	r	P	Beta	P
Age	-0.130	0.200		
BMI	0.045	0.660		
Gender	0.114	0.261		
Thalassemia	0.387	< 0.001	0.783	0.032
Systolic BP	0.022	0.832		
Diastolic BP	-0.173	0.087		
Hemoglobin	0.324	0.001	-0.563	0.116
PLT	-0.199	0.048	-0.199	0.048
Ferritin	-0.298	0.016	-0.203	0.110

Table 5 summarizes results of correlation and regression analysis between MPI and clinical and hematological parameters. In our study, it was shown that there was a negative correlation between MPI and thalassemia major (r = -0.288; P = 0.004) and hemoglobin values (r = -0.286; P = 0.04).

**Table 5:** Bivariate Correlation and Multiple Linear Regression Analysis Between Myocardial Performance Index and Other Variables

Parameters	Bivariate analysis		Multiple linear regression analysis	
	r	P	Beta	P
Age	0.127	0.212		
BMI	-0.157	0.120		
Gender	0.115	0.258		
Thalassemia	-0.288	0.004	0.075	0.844
Systolic BP	0.004	0.966		
Diastolic BP	0.011	0.915		
Hemoglobin	-0.286	0.004	-0.353	0.342
PLT	0.196	0.052		
Ferritin	0.242	0.052		

## Discussion

In our study, it was shown that echocardiographic elasticity parameters such as MPI, aortic strain, aortic distensibility, aortic stiffness index and elastic modulus differed significantly in thalassemia patients without known cardiac dysfunction or symptom who had normal routine echocardiographic parameters such as EF when compared to controls.

Cardiovascular diseases are most important causes of mortality in beta-thalassemia syndromes. Parenchymal injury secondary to myocardial iron deposition is the most important pathological mechanism in the development of cardiovascular diseases [1]. Iron deposition initially occurs in ventricles, and then extends to atrium and cardiac conduction system is involved finally. Degree of cardiac involvement depends on amount of iron deposition per myocardial fibril and number of fibrils involved. Cardiac iron deposition results in hypertrophy, dilatation and myocardial fibrosis.

It is known that early ventricular myocardial dysfunction can occur even in patients with beta-thalassemia receiving effective chelation therapy. MPI is obtained from ratio of isovolumetric contraction and relaxation times to ejection time, which can assess both systolic and diastolic functions, and appears as a marker used for early identification of myocardial functions in many instances. In a study on diabetic patients without marked cardiac failure or coronary artery disease, authors concluded that MPI can be a sensitive marker for diagnosis of LV dysfunction in patient with diabetes mellitus [9].

Elevated MPI is associated with cardiac disorders such as heart failure. MPI is a readily available parameter that does not prolong echocardiographic evaluation time and is independent from ventricular geometry. It can be used by all clinicians due to low inter-observer variability [3]. In our study, it was shown that MPI was disrupted in disadvantage of the patients with beta-thalassemia major in whom routine echocardiographic parameters such as ejection fraction are normal. This finding supports the idea that MPI can be a good marker for demonstration of cardiac involvement, especially in asymptomatic patients with beta-thalassemia.

It is known that vascular pathologies as well as myocardial parenchymal injury facilitate cardiovascular complications in patients with thalassemia. It is also known that vascular complications such as early arterial aging and atherosclerosis are more commonly observed in patients with thalassemia [10]. In *in vitro* studies, it was shown that there was an increase in soluble adhesion molecules, decrease in endothelial cell mitosis, apoptosis and abnormality vascular endothelial cells when cells from healthy individuals were incubated by thalassemic serum [11].

Role of iron deposition in the development of atherogenesis-related disorders is not fully understood although its pathological role in myocardial injury is well known. It is known that there is an association between increased lipid peroxidation products and atherogenic vascular complications in patients with thalassemia major [12]. In a study by Cheung et al, it was shown that iron deposition leads endothelial dysfunction by reducing nitric oxide bioavailability in patients with beta-thalassemia major [13]. Although the role of iron deposition for increased lipid peroxidation products has not been fully elucidated, it is shown that primary mechanism is oxidative stress in vessel wall directly caused by iron and involvement of iron in free radical reactions [4, 14, 15]. It has been proposed that oxidized-LDL fragments resulting from interactions among iron, free oxygen radicals and LDL facilitate atherosclerosis development [16].

However, physiopathology of vasculopathy in patients with thalassemia cannot be explained by iron-related oxidative injury alone, as arterial stiffness or similar disorders does not always accompany to hemochromatosis. Hemolytic process resulting in release of free hemoglobin observed in the course of patient with thalassemia contributes to vasculopathy by impairing nitric oxide bioavailability and activity. However, high levels of inflammatory markers such as interleukin-6 and soluble vascular adhesion molecules have been linked to impaired endothelial function in patients with thalassemia [4]. Activation of endothelial cells facilitates atherogenesis in patients with thalassemia by increasing leukocyte adhesion and migration. In patients with thalassemia, structural changes in available mechanisms result in altered elasticity of vessels in subsequent period, thus, predispose to arterial dysfunction.

The term, arterial stiffness, is used to define viscoelastic features of vessel wall such as mechanical strain or elasticity. Arterial stiffness, used as one of the important mechanical parameters of cardiovascular system, is created by dynamic and complex interaction between cellular and structural elements within vessel wall [17]. In general, increased arterial stiffness, decreased arterial elasticity (distensibility) and strain seen in atherosclerosis are considered to be independent risk factors for cardiovascular complications [18].

There is remarkable evidence indicating that increased stiffness in large arteries is major determinant of adverse cardiovascular results [19]. Aortic stiffness increases systolic BP and left ventricular mass due to decreased buffering mechanism and rapid return of reflective pressure waves from periphery. Associated decrease in diastolic pressure predisposes to increased cardiovascular disorder by reducing coronary blood flow [20]. Arterial stiffness has predictive value in other vascular disorders such as renal disease, stroke and dementia in addition to being total mortality and cardiovascular morbidity [21].

In recent years, arterial elasticity parameters which can be assessed by relatively simple, non-invasive methods are used to assess vascular involvement in patient with beta-thalassemia. Increased aortic stiffness or decreased distensibility is used as a marker for diffuse atherosclerotic involvement in vascular system [22]. Moreover, in recent studies, it is suggested that arterial stiffness can be used as a marker for early cardiovascular and vascular involvement in asymptomatic thalassemia patients without significant iron load [23]. In a study by Strakos et al, it was shown that aortic stiffness was associated to increased left ventricular mass and LA dilatation in asymptomatic thalassemia patients without increased iron. Similarly, in a study by Gedikli et al, it was shown that aortic stiffness parameters could have significant association to ferritin values [17]. However, serum ferritin level can fail to show body iron status accurately in many conditions such as pregnancy, acute or chronic inflammatory disease, malignity, infection, renal failure and malabsorption syndrome. Likewise, Cheung et al showed that there was no correlation between aortic stiffness and ferritin in their study [24].

In our study, correlation and regression analyses between aortic stiffness and strain and clinical and hematological parameters showed that there was a positive correlation between aortic stiffness and platelet and ferritin values. In addition, it was shown that beta-thalassemia and platelet values played role as independent risk factor for aortic stiffness.

In conclusion, it is known that early cardiac dysfunction can develop in patients with beta-thalassemia, even in those receiving effective chelation therapies. MPI and arterial elasticity indices can help clinician for early detection of cardiovascular disorders in patients with thalassemia even in the presence of normal echocardiography findings such as ejection fraction. Particularly, the indices should be used to predict and monitor cardiovascular complications in asymptomatic patients with beta-thalassemia.

### Study limitations

One limitation is to use BP values obtained from brachial artery in the assessment of aortic elasticity, although the fact that BP was measured by using same technique in both thalassemia and control groups did not affect the results. In addition, another limitation is failure to use T2-weighted MRI as marker of body iron load.
